# Immune checkpoint inhibitors and myocarditis: Lessons from a nationwide cohort study

**DOI:** 10.18632/oncoscience.617

**Published:** 2025-05-02

**Authors:** Bishal Tiwari

**Affiliations:** ^1^Nassau University Medical Center, East Meadow, NY 11554, USA

**Keywords:** immune checkpoint inhibitors (ICIs), non-small cell lung cancer (NSCLC), ICI-associated myocarditis, cardiotoxicity, immunotherapy-related adverse events

Immune checkpoint inhibitors (ICIs) have revolutionized the management of advanced non–small cell lung cancer (NSCLC), delivering unprecedented survival gains in pivotal trials of nivolumab/ipilimumab and pembrolizumab/chemotherapy [1, 2]. However, their use has also unveiled rare but potentially fatal cardiovascular toxicities, most notably myocarditis. In their nationwide cohort study, Li et al. report a 7.4-fold increase in 1-year risk of myocarditis among ICI users versus non-users (HR 7.41; 95% CI 3.29–16.67), based on 55219 patients drawn from China’s National Anti-Tumor Drug Surveillance System (NATDSS) between 2013 and 2021 [3]. This large-scale real-world evidence significantly advances our understanding of ICI-related cardiotoxicity and carries important clinical implications.

## Strengths and rigor of study design

Li et al. overcome many limitations of earlier reports by employing a new-user design, exposure density sampling to mitigate immortal-time bias, and time-dependent Cox models stratified by birth cohort. Their inclusion of 33 myocarditis events among 11213 ICI initiators and 28 events among 44006 non-users affords substantially greater statistical power than prior observational investigations [4]. Moreover, the comprehensive linkages to death registries, laboratory systems, and imaging archives within NATDSS lend robustness to outcome ascertainment, while multiple sensitivity analyses including competing-risk regression and active comparator analyses with targeted therapies consistently reinforce the primary findings [3].

## Clarifying the clinical spectrum and timing

Previous case series and pharmacovigilance data suggested an early onset of ICI-associated myocarditis typically within three months of therapy initiation with mortality rates approaching 50% [5]. Li et al. confirm a median time to myocarditis of 59 days in ICI users and reveal that 36% of events occur beyond the three-month window, indicating a more protracted risk period than previously appreciated. Their adjusted Kaplan–Meier estimates (4.8 vs. 0.6 per 1000 person-years) underscore that, although myocarditis remains uncommon, vigilant surveillance must extend at least six months post-ICI initiation [3].

## Contextualizing inconsistent meta-analytic findings

Meta-analyses of randomized trials have yielded conflicting conclusions regarding ICI-myocarditis risk, with one analysis of 24156 patients finding no significant association (RR 1.11; 95% CI 0.64–1.92) [6], and another reporting a significant Peto’s OR of 4.42 (95% CI 1.56–12.50) across 9 455 participants [7]. These disparities likely reflect selective trial populations, short follow-up, and underreporting of subclinical cases. The real-world cohort presented by Li et al. bridges this evidence gap, demonstrating a clear association in routine practice and highlighting the value of large observational datasets for rare toxicities.

## Mechanistic insights and biomarker development

Pathophysiologically, ICI-related myocarditis arises from unchecked T-cell–mediated injury to cardiac myocytes after blockade of CTLA-4 and PD-1/PDL-1 checkpoints [8]. Histopathologic confirmation of CD4+/CD8+ lymphocytic infiltrates and recent identification of autoreactive α-myosin–specific T cells further elucidate these processes [9]. Early data suggest that baseline troponin elevation or pre-existing autoantibodies may portend higher cardiotoxicity [10]. Li et al.’s findings reinforce the need to integrate cardiac biomarkers (troponin, natriuretic peptides), advanced imaging (CMR), and perhaps emerging immunophenotyping assays into ICI monitoring protocols to facilitate earlier detection and intervention.

## Limitations and areas for further study

Despite its strengths, the study relies on ICD-10 coding and diagnostic text mining, which may underestimate subclinical or mild myocarditis. The inability to grade severity, adjudicate cause of death, or distinguish among ICI regimens (e.g., CTLA-4 vs. PD-1/PD-L1 combinations) constrains actionable insights into risk stratification. Additionally, residual confounding by unmeasured factors such as radiotherapy exposure, EGFR/ALK status, or preexisting subclinical cardiac disease cannot be fully excluded, though the computed E-values suggest a high threshold for such bias.

## Implications for practice and research

Clinicians should counsel patients regarding myocarditis risk, implement baseline and serial cardiac evaluations for at least six months following ICI initiation, and maintain a low threshold for cardiology referral upon symptom onset. Prospective studies are needed to compare myocarditis incidence across ICI combinations and explore dose-response relationships. Finally, translational research into predictive biomarkers and prophylactic strategies perhaps leveraging immunomodulatory agents or novel checkpoint targets will be essential to optimize the risk benefit balance of ICIs in NSCLC and other malignancies.

## CONCLUSIONS

Immune checkpoint blockade exemplifies precision oncology’s promise and perils. Li et al. provide compelling, real-world evidence that advances our understanding of ICI-associated myocarditis in advanced NSCLC. Their rigorous methodology and comprehensive analyses should inform guideline development and stimulate further investigation into mechanisms, biomarkers, and preventive strategies for this serious immune-related toxicity.
